# Glutaminase isoforms expression switches microRNA levels and oxidative status in glioblastoma cells

**DOI:** 10.1186/s12929-021-00712-y

**Published:** 2021-02-20

**Authors:** Juan de los Santos-Jiménez, José A. Campos-Sandoval, Clara Márquez-Torres, Nieves Urbano-Polo, David Brøndegaard, Mercedes Martín-Rufián, Carolina Lobo, Ana Peñalver, María C. Gómez-García, Janet Martín-Campos, Carolina Cardona, Laura Castilla, Felipe da Costa Souza, Tzuling Cheng, Juan A. Segura, Francisco J. Alonso, Rui Curi, Alison Colquhoun, Ralph J. DeBerardinis, Javier Márquez, José M. Matés

**Affiliations:** 1 Departamento de Biología Molecular y Bioquímica and Instituto de Investigación de Biomedicina de Málaga (IBIMA), Universidad de Málaga, Málaga, Spain; 2 Department of Cell and Developmental Biology, Biomedical Sciences Institute, University of São Paulo, São Paulo, Brazil; 3 IDEAYA Biosciences, South San Francisco, CA USA; 4 Interdisciplinary Post-Graduate Program in Health Sciences, Cruzeiro do Sul University, São Paulo, Brazil; 5 Children’s Medical Center Research Institute, University of Texas Southwestern Medical Center (UTSMC), Dallas, TX USA; 6 Department of Pediatrics, UTSMC, TX Dallas, USA; 7 McDermott Center for Human Growth and Development, UTSMC, Dallas, TX USA

**Keywords:** Antioxidant enzymes, Cancer, Glioblastoma, Glutaminase, microRNA, Oxidative stress

## Abstract

**Background:**

Glutaminase isoenzymes GLS and GLS2 play apparently opposing roles in cancer: GLS acts as an oncoprotein, while GLS2 (GAB isoform) has context specific tumour suppressive activity. Some microRNAs (miRNAs) have been implicated in progression of tumours, including gliomas. The aim was to investigate the effect of GLS and GAB expression on both miRNAs and oxidative status in glioblastoma cells.

**Methods:**

Microarray profiling of miRNA was performed in GLS-silenced LN229 and GAB-transfected T98G human glioblastoma cells and their wild-type counterparts. Results were validated by real-time quantitative RT-PCR. Oxidative status and antioxidant enzymes were determined by spectrophotometric or fluorescence assays in GLS-silenced LN229 and T98G, and GAB-transfected LN229 and T98G.

**Results:**

MiRNA-146a-5p, miRNA-140-3p, miRNA-21-5p, miRNA-1260a, and miRNA-92a-3p were downregulated, and miRNA-1246 was upregulated when GLS was knocked down. MiRNA-140-3p, miRNA-1246, miRNA-1260a, miRNA-21-5p, and miRNA-146a-5p were upregulated when GAB was overexpressed. Oxidative status (lipid peroxidation, protein carbonylation, total antioxidant capacity, and glutathione levels), as well as antioxidant enzymes (catalase, superoxide dismutase, and glutathione reductase) of silenced GLS glioblastoma cells and overexpressed GAB glioblastoma cells significantly changed versus their respective control glioblastoma cells. MiRNA-1246, miRNA-1260a, miRNA-146a-5p, and miRNA-21-5p have been characterized as strong biomarkers of glioblastoma proliferation linked to both GLS silencing and GAB overexpression. Total glutathione is a reliable biomarker of glioblastoma oxidative status steadily associated to both GLS silencing and GAB overexpression.

**Conclusions:**

Glutaminase isoenzymes are related to the expression of some miRNAs and may contribute to either tumour progression or suppression through certain miRNA-mediated pathways, proving to be a key tool to switch cancer proliferation and redox status leading to a less malignant phenotype. Accordingly, GLS and GAB expression are especially involved in glutathione-dependent antioxidant defence.

## Background

Although metabolic profiles of tumours depend on both the genotype and tissue of origin, cancer cells have a very different metabolic profile compared to normal cells [1]. Glutaminase (GA; EC 3.5.1.2) catalyses the first step of glutaminolysis transforming glutamine into glutamate. Glutamate is one of the precursor amino acids for glutathione, which is present within the cell in a reduced (GSH) and an oxidized form (GSSG) [2]. The ratio of GSH/GSSG is the most important regulator of the cellular redox status, which together with metabolic stress switched by glutamine and GA can be a key regulatory tool to determine cell fate, leading to normal or tumour proliferation [3]. In humans, the GA family consists of two genes, *GLS* and *GLS2*, located in chromosome 2 and 12, respectively [2]. The *GLS* gene encodes two isoforms, known as kidney (K-type) glutaminase or KGA, and a shorter spliced form named glutaminase C or GAC [4]. These two isoenzymes are usually referred to as GLS [3]. On the other hand, the *GLS*2 gene codes for the liver (L-type) isozymes, named LGA, as well as for a longer isoform named GAB originally described in breast cancer cells [2]. Both isoenzymes are collectively designated as GLS2 [3].

MiRNAs are a family of 19- to 25-nucleotides noncoding small RNAs that primarily function as gene regulators suppressing mRNA translation. Aberrant miRNA expression has been described for several human malignancies, and this new class of small regulatory RNAs can have both oncogenic and tumour suppressor functions [5]. MiRNAs may interact with the 3’-UTRs of target mRNA molecules through partial sequence homology. They form an important class of regulators that participate in diverse biological functions including cell proliferation, differentiation and apoptosis. In fact, recent studies have shown that some miRNAs regulate the expression of several tumour suppressors and oncogenes and their dysregulation contributes to carcinogenesis [6]. It is now evident that changes in miRNA are involved in cancer progression, but the mechanisms of transcriptional regulation of miRNAs remain unknown [5]. To test if GLS isoenzyme expression is essential for miRNA regulation in glioblastoma (GBM) cells, an independent *GLS*-targeted shRNA was expressed in LN229 cells. We chose this cell line because it uses glutamine as the preferred anaplerotic precursor when given access to a physiological mixture of nutrients [7]. Furthermore, this cell line does not express appreciable amounts of the GLS2 isoforms LGA or GAB, increasing the likelihood of reducing cell growth by targeting *GLS* [7]. Additionally, stable transfection of GBM T98G cells with a vector carrying human full-length GAB sequence, T98G-GAB(+) [3], was carried out to ascertain the role of GAB in GBM cells, which has been linked to p53 pathway and antioxidant function [2, 3]. Hence, GLS2 increased cellular levels of GSH and NADH and decreases reactive oxygen species (ROS) levels in hepatocarcinoma cells [8]. Consistently, GLS2 protected these cells from DNA oxidation and ROS-sensitive apoptosis [9]. On the other hand, miRNA-200c induced ROS generation in ischemic cardiomyocytes through GA as GLS has been characterized as a direct target of miR-200c in this cell model [10]. Interestingly, long non-coding ribonucleic acid (lncRNA) urothelial carcinoma-associated 1 (UCA1) was highly expressed in bladder cancer cells. UCA1 regulates the expression of GLS2 by interfering with miR-16, and blocked ROS formation in bladder cancer [11]. In this study, we aim to elucidate whether some miRNAs can be modulated either by inhibition of GLS or by overexpression of GAB, impacting the redox state of cancer cells and contributing to the alterations in the markers of oxidative stress.

## Methods

### Cell lines, culture conditions and stable transfections

GLS-silenced LN229 and its control LN229 cells were established and characterized as described [7]. Both GLS isoforms KGA and GAC have been silenced as previously published by authors [3, 7]. Those cells were cultured in Dulbecco’s Modified Eagle Medium (DMEM) supplemented with 10 % foetal bovine serum (FBS), 100 I.U./mL penicillin, 100 µg/mL streptomycin, and 6 mM L-glutamine as previously described by authors [7]. In all stable knockdown experiments, very few detached cells were noted in the culture, and these were not included in growth and viability counts. T98G human GBM cell line was purchased from American Type Culture Collection (ATCC, Rockville, MD, USA). T98G-GAB(+) and control cells T98G-pcDNA were maintained in minimum essential medium supplemented with 10 % FBS, 1 % non-essential amino acids, and 100 I.U./mL penicillin and 100 µg/mL streptomycin. LN229-GAB(+) and control LN229-pcDNA human GBM cell lines were kindly provided by Monika Szeliga, Department of Neurotoxicology, Mossakowski Medical Research Centre, Polish Academy of Sciences, Warsaw, Poland. LN229-GAB(+) and LN229-pcDNA cell lines were maintained in DMEM as previously described for other LN229 cell lines [7]. GLS-silenced T98G and its control T98G were generated by transient silencing, using GLS-targeted small interferent RNAs (siRNAs) and a negative control siRNA transfection, respectively. Cells were cultured in DMEM as previously described for other T98G models. All cultures were maintained at 37 °C in a humidified atmosphere with 95 % air and 5 % CO_2_. T98G-GAB(+), T98G-pcDNA, LN229-GAB(+) and LN229-pcDNA cell lines were obtained by stable transfection of T98G or LN229 cells with the full-length cDNA sequence encoding human GAB isoform or empty pcDNA3 vector, respectively, as described previously [3, 12]. The culture medium for the polyclonal populations of T98G-GAB(+) and its control T98G-pcDNA, and for LN229-GAB(+) and its control LN229-pcDNA cells containing the neomycin-resistance gene was supplemented with 0.5 and 0.75 mg/mL geneticin, respectively. All cell lines were authenticated by both microscopic cellular morphology and growth curve analysis. All cell lines were also checked to be free of mycoplasma contamination.

### Protein expression

KGA and GAC downregulation was confirmed in the newly prepared cell line, T98G-GLS(−) by Western-blot (Additional file 1: Fig. S1). Cells were washed in PBS and harvested in lysis buffer (1 % NP-40, 150 mM NaCl, 50 mM Tris-HCl pH 8.0, supplemented with cOmplete^™^ Mini Protease Inhibitor, Roche, Indianapolis, IN, USA). Lysates were centrifuged at 12,000 g, 15 min at 4 °C. Supernatants were collected and stored at -80 °C. Protein quantification was performed using Pierce™ BCA Protein Assay Kit (ThermoFisher Scientific, Waltham, MA, USA). After heating 5 min at 95 °C, twenty micrograms of protein was resolved on 10 % polyacrylamide-SDS gels and then electrotransferred to nitrocellulose membranes. Membranes were blocked for 1 h at RT with 5 % non-fat milk in TBST. For GLS detection, two primary antibodies targeting specifically KGA or GAC isoforms were used, diluted in 5 % BSA in TBST and incubated overnight at 4 °C in agitation. These two GLS-targeted antibodies were kindly donated by Prof. Norman P. Curthoys (Colorado State University, CO, USA), both were generated in Rabbit again peptides containing the 20 C-terminal aminoacids of human KGA or GAC, respectively. Both antibodies were demonstrated to react specifically with its GLS isoform (KGA or GAC), and not to react with other glutaminase isoforms [3]. After incubation with respective primary antibodies, the membranes were washed in TBST and incubated 1 h RT with a commercial secondary HRP-conjugated Goat anti rabbit antibody (A0545, Merck) in 5 % BSA in TBST, and visualized in a ChemiDoc™ Gel Imaging System (Bio-Rad, Hercules, CA, USA), using an enhanced chemiluminiscence (ECL) detection system (SuperSignal™ West Pico, ThermoFisher Scientific). A commercial antibody targeting β-Actin (MAB1501, Merck) was used as a loading control, following the same procedure described before for the GLS antibodies, but using a Rabbit anti Mouse secondary HRP-conjugated antibody (A9044, Merck) in 5 % BSA in TBST (Additional file 1: Fig. S1a, c). For data quantification, Image Lab Software (Bio-Rad) was used, following the manufacturer’s instructions. In brief, each band was detected and selected, and background signal was descarted. After that, net values corresponding to the signal of each KGA or GAC band was normalized to β-Actin signal. For a more clear representation, the values obtained for T98G-GLS(−) and its T98G control were referred to the T98G control values, and the resulting ratio was represented (Additional file 1 : Fig. S1b, d).

### RNA interference and miRNA array experiments

All RNA interference (RNAi) experiments used pools of cells. Vectors for RNAi, lentiviral particles and details to obtain GLS knockdown LN229 cells have been described previously by authors [7]. Stably infected pools with adequate silencing were maintained with 1 µg/mL puromycin. LN229-GLS (−) express an shRNA targeting GLS, while its control LN229 cells contain the same construct but expressing green fluorescent protein (GFP) (LN229-GFP) instead of the GLS-targeted shRNA. GLS silencing in T98G cells were carried out by transfection using Lipofectamine™ 3000 reagent (Invitrogen, Grand Island, NY, USA) with GLS-targeted siRNAs, using a 1:1 mixture of two commercial siRNAs targeting GLS (NM_014905, sequence start around 1072 and 780, Merck KGaA, Darmstadt, Germany).

For miRNA expression analysis of different GBM cell lines, RNA was extracted using standard RNA extraction protocols (Trizol^™^, Invitrogen, Grand Island, NY, USA) according to manufacturer’s instructions. Four vials containing the GBM cell samples were shipped to Miltenyi Biotec, Bergisch Gladbach (Germany), on dry ice. RNA quality was assessed with the Agilent 2100 Bioanalyser (Agilent Technologies, Palo Alto, CA). Samples included in the present analysis had RNA Integrity Numbers (RIN) between 9.8 and 10.0 (Additional file 2: Fig. S2a, b). Labelling and hybridization were performed according to user manuals of the miRXplore™ instrument (Miltenyi Biotec). In brief, 1.2 µg/sample total RNA were labelled with the red fluorescent Hy5 using the miRNA/LNA labelling Exiqon kit. A pool of synthetic miRNAs in equimolar concentrations was designed by Miltenyi based on sequences of miRBase 9.2 and labelled with Hy3 (Additional file 3: Table S1). Subsequently, the labelled material was hybridized overnight to miRXplore™ microarrays using the a-Hyb™ Hybridization Station (Miltenyi Biotec). Fluorescence signals of the hybridized miRXplore™ microarrays were detected using ImaGene® software (Biodiscovery, Hawthorne, CA, USA) and raw data were acquired with PIQOR™ analyser software (Miltenyi Biotec), as detailed in Additional file 2: Fig. S2c, d.

### RNA isolation and real‐time quantitative reverse transcription-PCR

Total RNA was isolated from cultured cells, using miRCURY RNA isolation kit (Qiagen, Hilden, Germany) according to the manufacturer’s instructions. Twelve miRNAs were selected from the miRNA microarray analysis (see Table 1). Only the RNA samples with 260/280 ratios of 1.8–2.0 were used for further investigation. cDNA was generated from 1 µg total RNA using the miRCURY LNA Universal RT microRNA PCR, Universal cDNA Synthesis Kit II (Qiagen). Real-time quantitative reverse transcriptase-polymerase chain reaction (real-time qRT-PCR) was performed using the miRCURY LNA Universal RT microRNA PCR, ExiLENT SYBR® Green PCR Kit (Qiagen) according to the manufacturer’s instructions, using CFX96 Real-Time System C1000 Thermal Cycler (Bio-Rad). Oligonucleotides sequences are shown in Table 1. Samples that lacked either a template or reverse transcriptase were used as controls. To normalize the data for quantification of miRNAs, U6 small nuclear RNA was selected as the best endogenous control. Control LN229 and T98G cell lines were used as the miRNA expression level references. Real-time qRT-PCR amplifications for the miRNAs and U6 snRNA were performed after an initial denaturation step of 10 min at 95 °C followed by 40 cycles of 10-sec denaturation at 95 °C and 1-min annealing/extension at 60 °C. The relative expression of each miRNA was calculated according to the comparative 2^−ΔΔC*t*^ quantification method where ΔC_*t*_=C_*t*_(sample) − C_*t*_(normaliser) and ΔΔC_*t*_ = ΔC_*t*_(sample)−ΔC_*t*_(calibrator). The control U6 reactions were implemented with 5′-CTCGCTTCGGCAGCACA-3′ as the forward primer and 5′-AACGCTTCACGAATTTGCGT-3′ as the reverse primer. Standard deviation (SD) was incorporated into fold change by expressing the fold change as a range. This is done by incorporating the SD into the formula: 2 − ΔΔCt + SD and 2 − ΔΔCt – SD.

**Table 1:** Selected microRNAs

Name	Forward primer (5′-3′)	Human chromosome	Gene name	Gene region	*GLS* hybridization	*GLS2* hybridization
microRNA-21-5p	TAGCTTATCAGACTGATGTTGA	17q23.1	*VMP1*	UTR	330 and 949	
microRNA-92a-3p	TATTGCACTTGTCCCGGCCTGT	13q31.3	*LINCRNA48*	Intron, UTR		
microRNA-140-3p	TACCACAGGGTAGAACCACGG	16q22.1	*WWP2*	Intron		
microRNA-146a-5p	TGAGAACTGAATTCCATGGGTT	5q34	*PTTG1*	UTR		175
microRNA-203a	GTGAAATGTTTAGGACCACTAG	14q32.33			93	
microRNA-363-3p	AATTGCACGGTATCCATCTGTA	Xq26.2				
microRNA-487b-5p	GTGGTTATCCCTGTCCTGTTCG	Xq26.2	*AL132709.4*	Intron		
microRNA-663a	AGGCGGGGCGCCGCGGGACCGC	20p11.1				29
microRNA-762	GGGGCTGGGGCCGGGGCCGAGC	16p11.2	*BCL7C*	Exon	1817	
microRNA-1246	AATGGATTTTTGGAGCAGG	2q31.1			566	45
microRNA-1260a	ATCCCACCTCTGCCACCA	14p24.3	*NGB*	Exon		383
microRNA-1274a	TTCAGGTCCCTGTTCAGGCGCC	5p13.1	*PLCXD3*	Intron		

Second column shows the primers used for targets amplification. Human karyotypic location of respective microRNAs were obtained from mirbase database (http://www.mirbase.org/). MicroRNAs respective sequences are present in some genes: name and location were determined using CoGemIR database (http://cogemir.tigem.it/), and hybridization with *GLS* and *GLS2* genes were obtained using miRanda database (http://cbio.mskcc.org/microrna_data/miRanda-aug2010.tar.gz), and RNAhybrid (http://bibiserv.techfak.uni-bielefeld.de/rnahybrid/). VMP1: Vacuole Membrane Protein 1; LINCRNA48: Long Intergenic Non-Protein Coding RNA 48; WWP2: WW domain containing E3 ubiquitin Protein ligase 2; PTTGA: Pituitary Tumour-Transforming Gene 1; BCL7C: BCL Tumour Suppressor 7 C; NGB: Neuroglobine; PLCXD3: Phosphatidylinositol specific phospholipase C X Domain containing 3

### GSH assays

The level of total GSH in every cell line was determined by using glutathione assay kit obtained from Cell Biolabs INC, San Diego, California, USA (#STA-312-T), according to the manufacturer’s instructions. Briefly, approximately 3.5 × 10^6^ cells were resolved. Supernatant samples were transferred to a microplate and added 25 µL of glutathione reductase (GR), 25 µL of NADPH solution, 100 µL of cell lysate, and 50 µL of assay buffer with 5,5′-dithiobis-(2-nitrobenzoic acid) (DTNB), reading every 60 s, during 10 min, at 405 nm. Absorbance was determined using a microplate spectrophotometer Eon, Biotek Instruments, Inc. (Winooski, VT, USA). All samples were run in duplicate. Total GSH amounts were normalized by the protein content (Bradford) in each sample, and a ratio was calculated by dividing the experimental values by the control values for each independent experiment.

GR activity was measured using the Glutathione Reductase Assay kit from Cell Biolabs, INC. (#STA-812), as described by suppliers. In brief, approximately 3.5 × 10^6^ cells were used for each experiment. Then, 25 µL of NADPH solution was added to a well in the 96 well plate and then 100 µL of cell lysate, 25 µL of GSSG, and 50 µL of assay buffer containing DTNB were added to each well. The absorbance was read at 405 nm every 1 min for 10 min in a microplate spectrophotometer Eon, Biotek Instruments, Inc. to calculate the activity. All samples were run in duplicate. GR activities were normalized by the protein content (Bradford) in each sample, and a ratio was calculated by dividing the experimental values by the control values for each independent experiment.

### Measurement of antioxidant activities

Catalase (CAT) activity was measured using the Catalase Assay kit from Cell Biolabs, INC (#STA-341-T), according to the manufacturer’s instructions. In brief, approximately 3.5 × 10^6^ cells were used for each experiment. Then, 20 µL of cytosolic lysate was mixed with 50 µL of 12 mM H_2_O_2_ solution and incubated for 1 min at room temperature. The reaction was stopped by adding 50 µL of stop solution (containing sodium azide). Then, 5 µL out of the 120 µL reaction mixture was mixed with 250 µL of the colour reagent in a new microplate. After 50 min of incubation for colour development, the absorbance was measured at 520 nm in a microplate spectrophotometer Eon, Biotek Instruments, Inc. All samples were run in duplicate. CAT activities were normalized by the protein content (Bradford) in each sample, and a ratio was calculated by dividing the experimental values by the control values for each independent experiment.

Superoxide dismutase (SOD) activity was measured using a SOD assay kit (Cell Biolabs, INC, #STA-340-T), as described by suppliers. Approximately 3.5 × 10^6^ cells were used for each experiment. SOD activity was measured by assessing the degree of inhibition of the reduction of nitroblue tetrazolium by xanthine oxidase. One unit of activity was considered as the amount of protein that gives 50 % inhibition of the reaction. Briefly, the cell lysates were incubated in a 96-well plate and the absorbance at 490 nm was measured kinetically at 37 °C for 30 min. Absorbance was determined using a microplate spectrophotometer Eon, Biotek Instruments, Inc. All samples were run in duplicate. SOD activities were normalized by the protein content (Bradford) in each sample, and a ratio was calculated by dividing the experimental values by the control values for each independent experiment.

### Oxidative stress assessment

Total antioxidant capacity (TAC) was determined using Cell Biolabs, INC kit (#STA-360-T), evaluating cupric reducing antioxidant capacity, based on the complex equilibria between Cu(II)–bathocuproinedisulfonic acid and Cu(I)–bathocuproinedisulfonic acid. Briefly, 3.5 × 10^6^ cells were used for each experiment. Then, 20 µL of cell lysate was loaded into a well in the 96 well plate and added 180 µL of reaction mix. Absorbance was quantified at 490 nm before adding 50 µL of Cu(II) solution to each well. Samples were incubated 5 min at RT; then, redox reactions were halted with 50 µL of a stop solution and final absorbance was measured. Absorbance was determined using a microplate spectrophotometer Eon, Biotek Instruments, Inc. All samples were run in duplicate. TAC values were calculated as nmol Copper Reducing Equivalents (CRE) and normalized by the protein content (Bradford) in each sample, and then a ratio was calculated by dividing the experimental values by the control values for each independent experiment.

Lipid peroxidation was evaluated by thiobarbituric acid reactive substances (TBARS) method, using Cell Biolabs, INC kit (#STA-330-T), which is based on malondialdehyde (MDA) reaction with TBARS, producing a pink complex with a peak absorbance at 532 nm. To ensure that no lipid oxidation occurs during the assay, butylated hydroxytoluene was added to the sample prior to lysis. Approximately 3.5 × 10^6^ cells were used for each experiment. Cell lysates were used to measure lipid peroxidation level according to the manufacturer’s instruction. Briefly, 100 µL of cell lysate were treated with reagents during 1 h at 95 °C, cooled on ice for 5 min, centrifuged at 1000 g for 15 min and used for measurement in a 96-well plate. Absorbance was determined at 532 nm using a microplate spectrophotometer Eon, Biotek Instruments, Inc. All samples were run in duplicate. TBARS values were normalized by the protein content (Bradford) in each sample and a ratio was calculated by dividing the experimental values by the control values for each independent experiment.

Protein carbonylation was performed as described by Cell Biolabs, INC kit (#STA-307-T) using a fluorescein 5-thiosemicarbazide (FTC)-based method. Approximately 7 × 10^6^ cells were used for each experiment. In brief, 50 µL of cell lysate was mixed with 50 µL of FCT solution. Proteins were precipitated by the addition of 4-volumes of cold 20 % trichloroacetic acid. Following 10 min incubation on ice, tubes were centrifuged at 9000 g for 10 min at 4 °C. Supernatants were decanted; precipitates were washed 3 times by vortexing with 1 mL of acetone. Finally, acetone supernatant was carefully decanted out and protein precipitates were air dried, solubilized with 50 µL of 6 M guanidine hydrochloride and diluted by the addition of 450 µL of pH 7.0 buffer. Protein concentration in each of these samples was measured by Bradford assay. The samples were aliquoted 100 µL per well in triplicate, and fluorescence measured in a microplate fluorescence reader FL600, Biotek Instruments, Inc., with excitation at 480 nm and emission at 530 nm. Fluorescence readings from 6 wells for each sample (3 for each of the duplicates) were averaged and nanomoles of FTC-reacted carbonyls were calculated using a standard curve generated from the readings of various concentrations of FTC prepared in a medium similar to that of the samples. Values were normalized to the protein content and expressed as pmol carbonyl/mg protein, and finally a ratio was calculated by dividing the experimental values by the control values for each independent experiment.

### Statistical analysis

For array and real-time qRT–PCR experiments, miRNAs with > 10 % undetectable expression values were excluded from the study. Statistical analysis was performed using software *Statgraphics*. Values were expressed as means ± SEM. Differences between groups were calculated with the Student’s t-test or Mann–Whitney U test. In addition, Spearman’s rank-order correlation test was performed. All results were considered significantly as p < 0.05. Experiments were performed at least three times.

## Results

### Up- and downregulation of miRNAs following GLS silencing or GAB overexpression

**Fig. 1:**
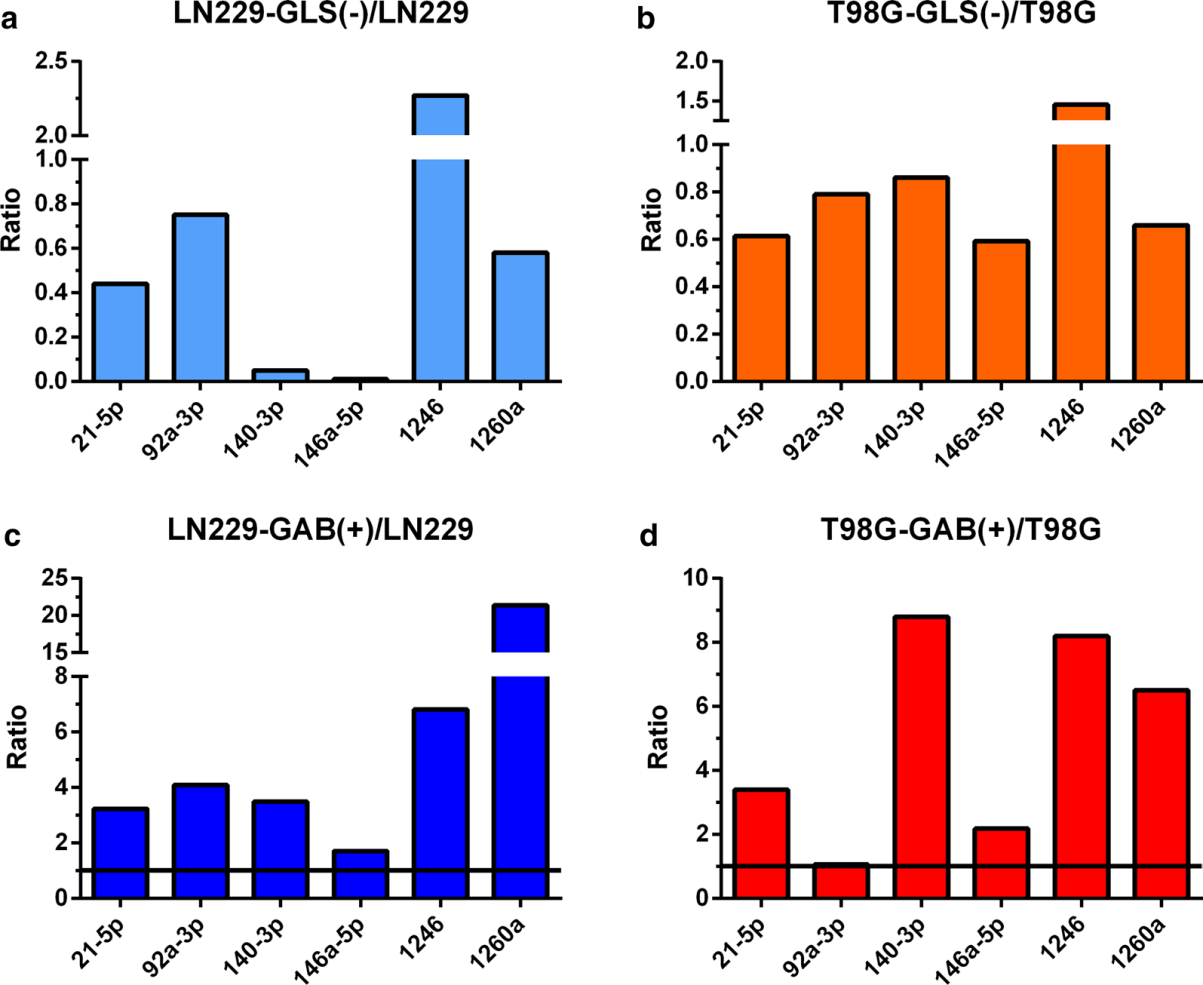
Down- and up-regulated miRNAs. Results are represented for LN229 (LN229-GFP) and LN229-GLS(−) cells (**a**), T98G (scrambled-transfected T98G) and T98G-GLS(−) (**b**), LN229 (LN229-pcDNA) and LN229-GAB(+) (**c**) and for T98G (T98G-pcDNA) and T98G-GAB(+) cells (**d**). The relative expression of each miRNA was calculated according to ΔΔCt method. Vertical coordinates represent the average fold change

The miRXplore™ chips enabled examination of 993 human miRNAs (Additional file 4: Table S2). To investigate the role of GLS and GAB isoforms in miRNAs regulation, we first selected the 23 miRNAs that strongly changed their expression levels when *GLS* was silenced or GAB was overexpressed (Table 2). Then, we analysed these profiles to find eight miRNAs (miRNA-21-5p, miRNA-92a-3p, miRNA-140-3p, miRNA-146a-5p, miRNA-762, miRNA-1246, miRNA-1260a, and miRNA-1274) which were highly under- or overexpressed in both models (GLS silenced cells or cells overexpressing GAB). In LN229-GLS(−) vs. LN229-GFP cells, thirteen miRNAs demonstrated enhanced overexpression, highlighting miRNA-1246 that showed 2.83-fold augmented expression. Among five downregulated miRNAs, miRNA-146a showed the highest reduced expression: 0.21-fold in these GLS silenced cells when compared to control LN229-GFP cells (Table 2). In T98G-GAB(+) compared to T98G-pcDNA control cells, 13 miRNAs showed higher expression, including miRNA-140-3p, miRNA-92a-3p, miRNA-1260, miRNA-21, miRNA-1246, and miRNA-146a-5p (Table 2). Only miRNA-762, miRNA-1246, and miRNA-1274 were similarly expressed in both models. The results from the microarray analysis were validated by real-time quantitative RT-PCR, as described in "Methods" section. From the twelve selected miRNAs (Table 1), six were analysed in two GBM cell models: LN229 and T98G cells. Both models were studied when GLS was silenced and when GAB was overexpressed (Fig. 1): LN229-GLS(−) cells vs. control LN229-GFP, T98G-GLS(−) cells vs. control (scramble siRNA-transfected) T98G, LN229-GAB(+) cells vs. control LN229-pcDNA, and T98G-GAB(+) cells vs. control T98G-pcDNA. Five miRNAs (miRNA-146a-5p, miRNA-140-3p, miRNA-21-5p, miRNA-1260a, and miRNA-92a-3p) were significantly repressed in LN229-GLS(−) vs. LN229-GFP cells, whilst miR-1246 was 2.3-fold upregulated in LN229-GLS(−) vs. LN229-GFP cells (Fig. 1a). Similar results were found comparing T98G-GLS(−) vs. control T98G cells, but miR-1246 was 1.5-fold upregulated in T98G-GLS(−) vs. control T98G cells (Fig. 1b). All six miRNAs assayed were overexpressed in LN229-GAB(+) cells (miRNA-21-5p, miRNA-92a-3p, miRNA-140-3p, miRNA-146a-5p, miRNA-1246 and miRNA-1260a) as stated in Fig. 1c. Five miRNAs: miRNA-140-3p, miRNA-1246, miRNA-1260a, miRNA-21-5p, and miRNA-146a-5p, were all significantly overexpressed in T98G-GAB(+) vs. control T98G-pcDNA cells. No significant differences were found for miRNA-92a-3p in this cell model (Fig. 1d).

**Table 2:** MicroRNA microarray was utilized to analyse 993 human microRNAs

microRNA	T98G-GAB(+)/T98G-pcDNA	LN229-GLS(−)/LN229-GFP
microRNA-21-5p	1.66	0.76
microRNA-22	n.d.	1.58
microRNA-27b	n.d.	1.56
microRNA-29c	n.d.	1.56
microRNA-30	2.10	n.d.
microRNA-92a-3p	1.93	0.68
microRNA-93	n.d.	1.67
microRNA-140-3p	2.16	0.50
microRNA-146a-5p	1.63	0.21
microRNA-203a	n.d.	1.87
microRNA-320c	1.86	n.d.
microRNA-363-3p	n.d.	1.98
microRNA-487b-5p	n.d.	2.85
microRNA-493	1.64	n.d.
microRNA-503-5p	n.d.	0.44
microRNA-548	n.d.	1.68
microRNA-638	n.d.	1.76
microRNA-663a	2.05	n.d.
microRNA-762	1.74	2.09
microRNA-1246	2.20	2.83
microRNA-1260a	1.84	0.78
microRNA-1274a	1.84	1.57
microRNA-1973	1.62	n.d.

*GFP* green fluorescent protein; Ratio of differentially expressed (> 1.5-fold or < 0.8-fold) microRNAs are depicted

As stated in "Background" section, miRNAs over- or under-expression has been associated with GLS [10] or GLS2 [11] expression levels, which are related with antioxidant capacity [2, 8, 9]. Following, we have measured a number of oxidative stress biomarkers in our GBM cell models that up- or down-express GA isoenzymes.

### Glutathione-dependent antioxidant capacity is an associated-biomarker to GLS and GAB expression

**Fig. 2:**
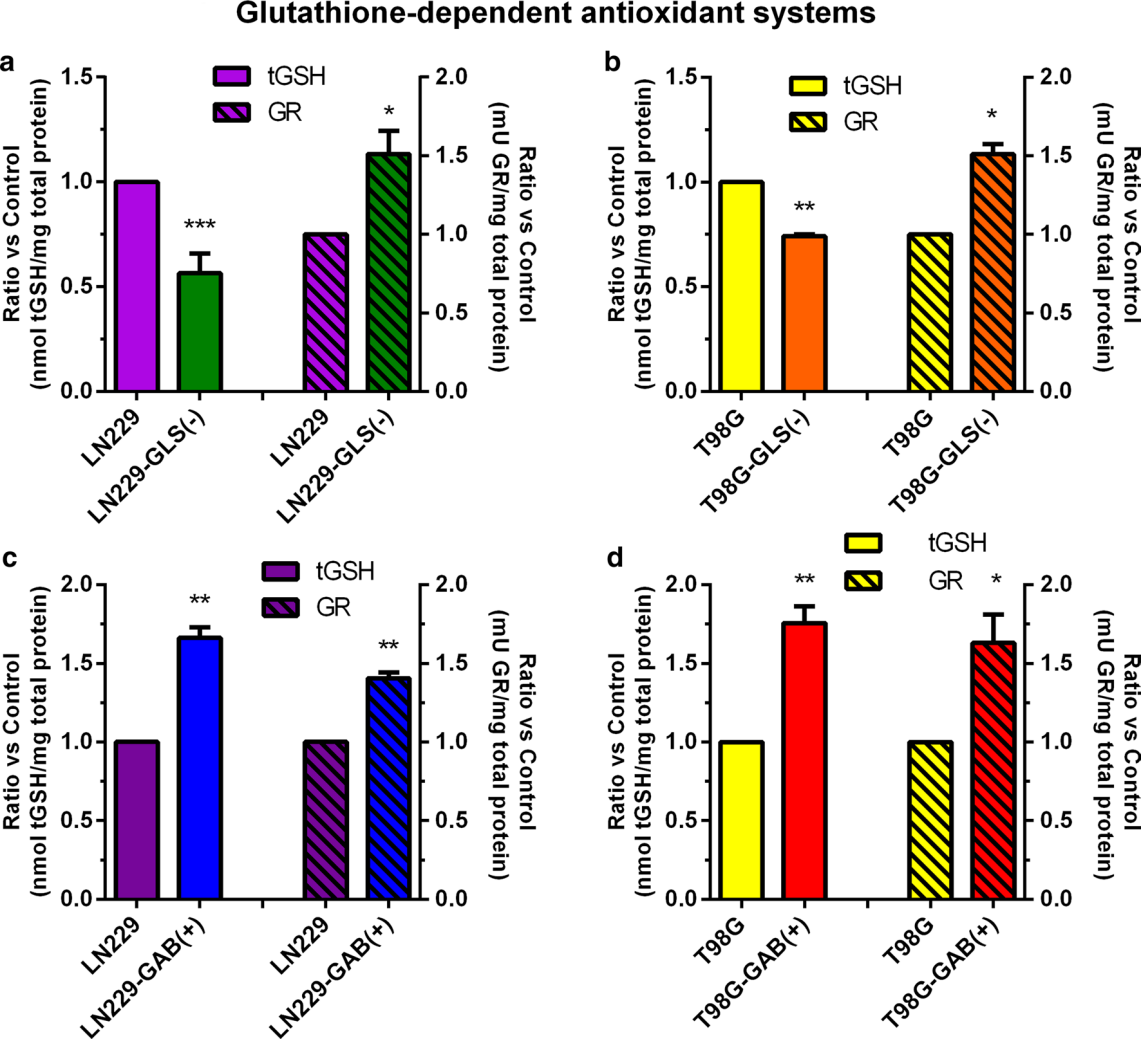
Total glutathione levels and GR activity. Results are represented for LN229 (LN229-GFP) and LN229-GLS(−) cells (**a**), T98G (scrambled-transfected T98G) and T98G-GLS(−) (**b**), LN229 (LN229-pcDNA) and LN229-GAB(+) (**c**) and for T98G (T98G-pcDNA) and T98G-GAB(+) cells (**d**). The results were calculated as nmoles GSH/mg protein, and mU/mg protein, respectively, and a ratio was calculated by dividing the values of the experimental sample by those of its control for each biological replicate. Data are expressed as mean ± S.E.M. (N = 3) of the ratios calculated for at least three independent experiments. *p < 0.05, **p < 0.01, ***p < 0.001, compared to the respective control group. *GR* glutathione reductase, *tGSH* total glutathione

When GLS was silenced total glutathione levels were reduced by 45 % in LN229-GLS(−) vs. LN229-GFP cells. Simultaneously, GR activity was increased by 50 % (Fig. 2a). Almost identical results were found comparing T98G-GLS(−) vs. T98G cells (Fig. 2b). When GAB was overexpressed in LN229 cells, glutathione levels were augmented 1.5-fold and GR activity was also significantly increased (Fig. 2c). Similar significant results were found for both GSH-dependent oxidative biomarkers when GAB was overexpressed in T98G cells (Fig. 2d).

### Catalase and superoxide dismutase activities change following both GLS silencing and GAB overexpression

**Fig. 3:**
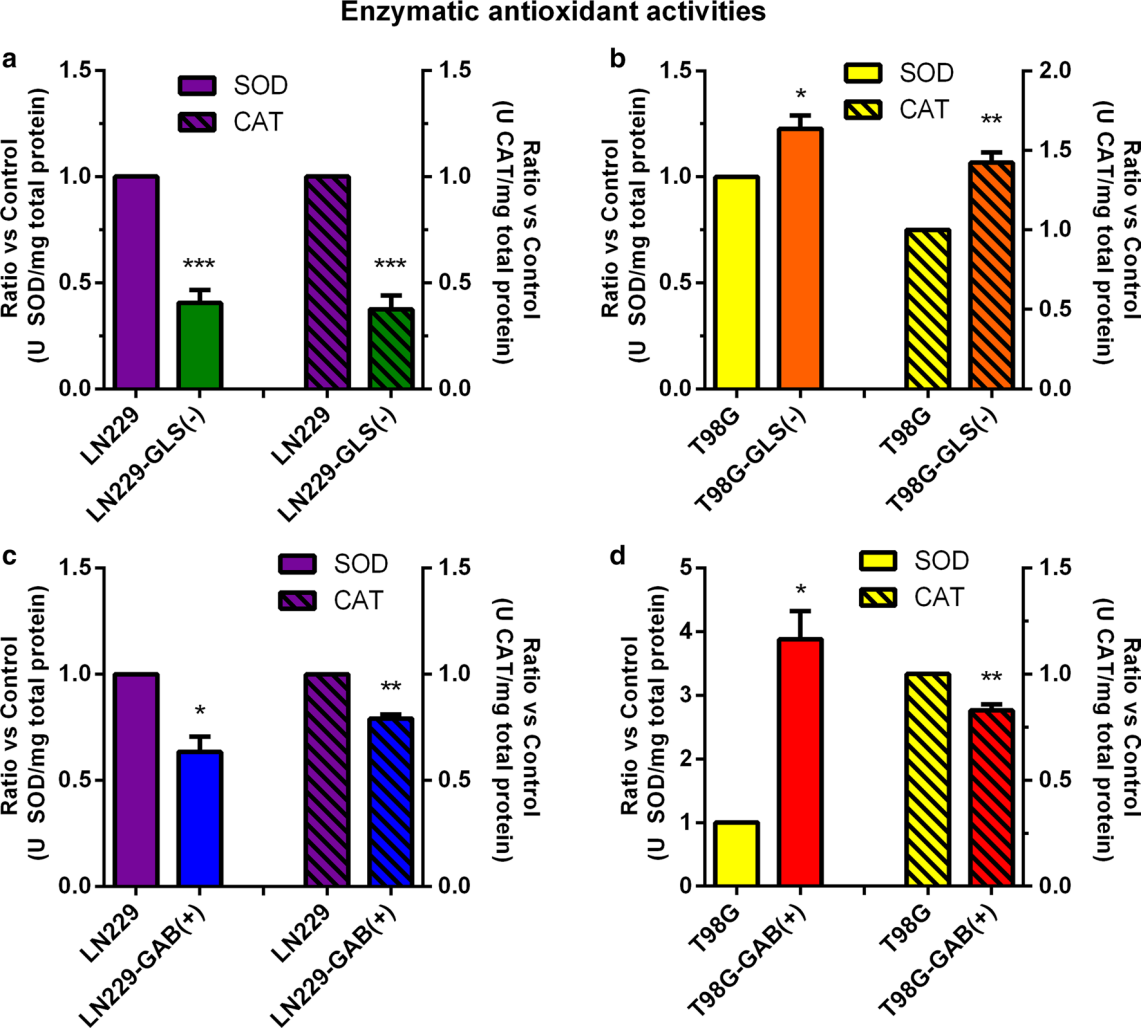
Antioxidant enzymatic activities. SOD and CAT were determined, for LN229 (LN229-GFP) and LN229-GLS(−) cells (**a**), T98G (scrambled-transfected T98G) and T98G-GLS(−) (**b**), LN229 (LN229-pcDNA) and LN229-GAB(+) (**c**) and for T98G (T98G-pcDNA) and T98G-GAB(+) cells (**d**). The results are expressed as U/mg protein and a ratio was calculated by dividing the values of the experimental sample by those of its control for each biological replicate. Data are expressed as mean ± S.E.M. (N = 3) of the ratios calculated for at least three independent experiments. **p < 0.01, ***p < 0.001, compared to the respective control group. *CAT* catalase, *SOD* superoxide dismutase

When GLS was silenced both CAT and SOD activities were significantly reduced in LN229-GLS(−) vs. LN229-GFP cells (Fig. 3a). However, CAT and SOD activities were both significantly enhanced when GLS was silenced in T98G cells (Fig. 3b). Decreased CAT and SOD activities were found when GAB was overexpressed in LN229 cells (Fig. 3c). SOD activity was significantly increased when GAB was overexpressed in T98G cells; however, CAT activity was slightly reduced (Fig. 3d).

### Lipid peroxidation was attenuated by both GLS silencing and GAB overexpression. TAC decreased by GLS silencing and increased when GAB was overexpressed

**Fig. 4:**
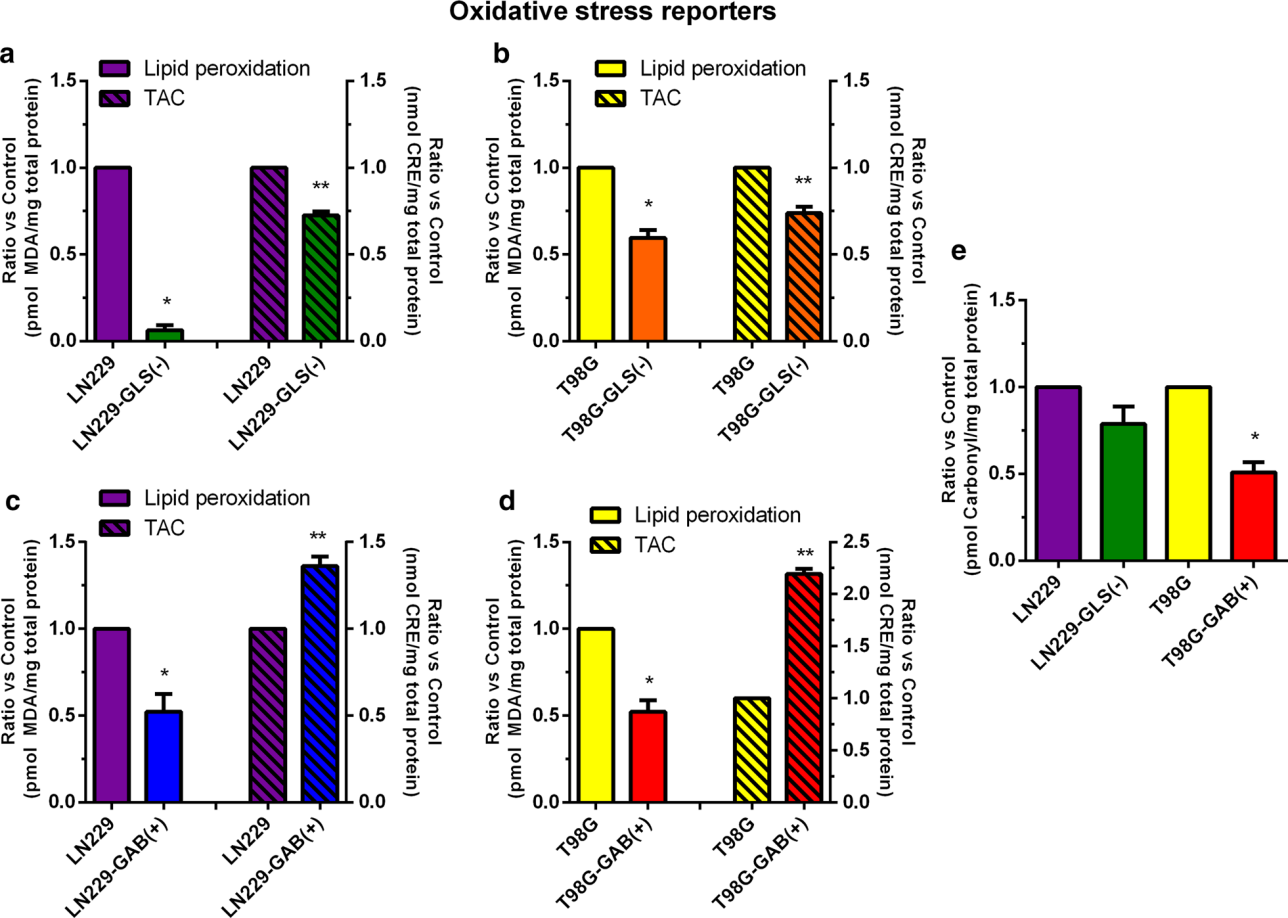
Oxidative status values. Lipid peroxidation and TAC are represented for LN229 (LN229-GFP) and LN229-GLS(−) (**a**), T98G (scrambled-transfected T98G) and T98G-GLS(−) (**b**), LN229 (LN229-pcDNA) and LN229-GAB(+) **(c)** and for T98G (T98G-pcDNA) and T98G-GAB(+) cells (**d**). Protein damage are depicted for LN229 (LN229-GFP) and LN229-GLS(−) cells and for T98G (T98G-pcDNA) and T98G-GAB(+) cells (**e**). The results are expressed as nmol CRE/mg protein, pmol MDA/mg protein, and pmol carbonyl/mg protein, respectively, and a ratio was calculated by dividing the values of the experimental sample by those of its control for each biological replicate. Data are expressed as mean ± S.E.M. (N = 3) of the ratios calculated for at least three independent experiments. *p < 0.05, **p < 0.01, ***p < 0.001, compared to the respective control group. *CRE* Copper Reducing Equivalents, *MDA* malondialdehyde, *TAC* total antioxidant capacity

Lipid peroxidation fell 10-fold in LN229-GLS(−) vs. LN229-GFP cells. In this model, TAC decreased to two thirds after GLS silencing (Fig. 4a). TAC trend was identical after GLS silencing in T98G model. Lipid peroxidation dropped almost to half in in T98G-GLS(−) vs. control T98G cells. (Fig. 4b). TAC was significantly increased and lipid oxidative damage, measured as TBARS, was significantly decreased when GAB was overexpressed in LN229 cells (Fig. 4c). Similar results were found when GAB was overexpressed in T98G cells (Fig. 4d). In addition, when GAB was overexpressed in T98G cells, protein damage declined to half. This oxidative stress parameter was 25 % reduced when GLS was silenced in LN229 cells (Fig. 4e).

## Discussion

Because cancer signalling pathways often exist in atypical forms, our understanding of cancer networks is currently not sufficient to allow *a priori* predictions of the cellular response [4]. However, combination therapies and sequential treatments to gain anticancer power are profusely recommended [7]. In GBMs, a thorough understanding of the vast genetic aberrations and signalling pathways involved in gliomagenesis as well as heterogeneous clinicopathological presentations remains elusive. The discovery of miRNAs and their capability of simultaneously regulating multiple downstream genes may play a key role in explaining the complex mechanisms underlying GBM formation [13]. Recent efforts against GBM malignancies have focused on therapies which target key intracellular apoptotic pathways which may confer tumour resistance, such as p53, and more recently some miRNA sequences [14]. Of particular interest in this research, direct repression of miRNA-23a and miRNA-23b by c-Myc is responsible for increased expression of GLS protein in human Burkitt lymphoma and prostate cancer [15]. In addition, miRNA-137 inhibits growth of malignant melanoma by targeting GLS [16]. On the other hand, expression of GLS2 by bladder cancer cells was regulated through interfering with miRNA-16 [11]. Furthermore, the capacity of GLS2 to alter miRNA patterns in cancer cells would be explained by recent findings showing that GLS2 is involved in miRNA regulation through Dicer stabilization [17]. As showed in Table 1, some of the sequences of the miRNAs up- or downregulated after changes in GLS or GLS2 expression are present in some genes that have been involved in glioma proliferation and inhibition of apoptosis as *NGB* [18], *WWP2* [19] and *PTTG1* [20]. Of interest, miRNA-1260a, miRNA-140-3p, miRNA-21-5p, and miRNA-146a-5p are among the validated by real-time qRT-PCR in each model with targeted GA expression, LN229-GLS(−), T98G-GLS(−), LN229-GAB(+) and T98G-GAB(+) (Fig. 1). On the other hand, miRNA-1246, which is the only that hybridize with both GLS(−) and GAB(+) transcripts (Table 1), was also the only miRNA overexpressed in all models, as shown in Fig. 1a–d.

Among miRNAs having notable expression differences between T98G-GAB(+) vs. T98G-pcDNA cells, and LN229-GAB(+) vs. LN229-pcDNA cells, miRNA-140-3p were increased 8.8- and 3.5-fold, respectively, as assessed by qRT-PCR. Although studies dealing with this particular miRNA in GBM or other brain tumours have not been yet published, our results showing a marked increase in the less-malignant and more differentiated GAB(+) cell line are in agreement with the downregulation of miRNA-140-3p found in lung cancer [21]. MiRNA-1246 huge expression (8.2- and 6.8-fold, respectively) in T98G and LN229 cells overexpressing GAB supports that this miRNA is present in less malignant human GBM cells [22]. MiRNA-1246 has been described as a novel interactor of p53 and its analogous p63 and p73, which are associated with apoptosis [23] and inhibition of growth in hepatocellular carcinoma (HCC) cells [23, 24]. Accordingly, miRNA-1246 targets p53, phosphatidylinositol-3-kinase/protein kinase B (PI3K/AKT), mitogen-activated protein kinase (MAPK), and mammalian target of rapamycin (mTOR) signalling pathways, leading to apoptosis in paclitaxel-treated HCC cells [23]. Of note, GAB has shown context-dependent tumour suppression activity as a target of the p53 family of tumour suppressors [2], and a recent work linked this GAB function with the negative regulation of PI3K/AKT pathway in hepatocellular carcinoma [25]. Thus, induction of miRNA-1246 after GAB overexpression may be one key mechanism to inhibit cancer progression through p53 and PI3K/AKT dependent pathways.

T98G and LN229 GAB overexpressing cells showed respectively a 6.5- and 21.4-fold overexpression of miRNA-1260a. Correspondingly, miRNA-1260a was up-regulated in the less proliferative atypical Spitz lesions relative to Spitz skin tumours [26]. MiRNA-21-5p was 3.4- and 3.2-fold expressed respectively in T98G and LN229 GBM cells overexpressing GAB. This miRNA targets the gene for p-21 protein, which is correlated with the anti-proliferative and cell death effects in T98G cells [27]. The tumour suppressing function of this miRNA was also characterized in thyroid papillary carcinoma via downregulation of Bcl-2 [28]. On the other hand, miRNA-146a-5p was 2.2- and 1.7-fold overexpressed in T98-GAB(+) and LN229-GAB(+) models, respectively. This is in accordance with its function as tumour suppressor in GBM by negatively regulating epidermal growth factor receptor (*EGFR*) and platelet-derived growth factor receptor beta (*PDGFRB*) genes [29]. MiRNA-146a-5p has been described as a native molecular brake for oncogenesis in aggressive and deadly brain tumours via NOTCH1 signalling [30]. It also functions as a tumour-suppressive miRNA targeting NOTCH2, inhibiting the epithelial-mesenchymal transition (EMT) progression of oesophageal squamous cell carcinoma (ESCC) [31]. Similarly, functional studies showed that this miRNA is a negative regulator of tumourigenic gene expression in microglia via its target *SMAD4* [32]. On the other hand, miRNA-146a-5p inhibits cervical cancer cell proliferation, migration and invasion, repressing the expression of histone demethylase [33]. Of note, miRNA-146a-5p has been found downregulated in prostate cancer cells treated with the anticancer agent metformin [34]. Finally, concerning miRNA-92a-3p, although no differences were found in T98G-GAB(+) vs. T98G-pcDNA cells, it was 4.1-fold overexpressed in LN229-GAB(+) vs. LN229-pcDNA cells. This data agrees with a very recent publication showing that miRNA-92a-3p overexpression is a potential biomarker of favourable outcome of chronic lymphocytic leukaemia patients [35]. Importantly, miRNA-92a-3p profile was markedly different (even or upregulated) from that downregulation described below for both LN229-GLS(−) vs. LN229-GFP cells and T98G-GLS(−) vs. control T98G cells (0.75-fold and 0.79-fold, respectively).

GBM LN229-GLS(−) cells dramatically repressed expression of miRNA-146a-5p. Its expression in T98G-GLS(−) was also decreased (0.6-fold). Although it is currently unknown whether miRNA-146a-5p possesses oncogenic properties in GBM cells, results obtained in breast cancer tissue and MCF-7 cell line support this role through the BRCA1 pathways [36]. These findings are in agreement with the role of miRNA-146a as an inflammatory miRNA that regulates the NF-κB pathway, transforming growth factor-β (TGF-β), and induces angiogenesis [37]. Noteworthy, miRNA-146a-5p has been recognized as a positive biomarker in lung cancer [38] and as an oncogenic factor in oral squamous cell carcinoma (OSCC). Diverse investigations have pointed out that miRNA-146a-5p affects proliferation and apoptosis in a cellular context-dependent manner, defining to this miRNA as a pleiotropic regulator of carcinogenesis [39].

LN229-GLS(−) showed 0.05-fold expression of miRNA-140-3p vs. LN229-GFP cells. However, in T98G-GLS(−) vs. control T98G cells its expression was only decreased 0.85-fold. Although not previous results have been published in GBM cells, miRNA-140-3p was up-regulated in other malignancies [40]. Versus LN229 and T98G, in both LN229-GLS(−) and T98G-GLS(−) miRNA-21-5p was underexpressed vs. their controls (0.44-fold and 0.60-fold, respectively). The oncogenic function of miRNA-21 in GBM cells was associated to the p53 circuit, targeting TGF-β, modulating cell growth, and repressing p53-mediated apoptosis in response to anticancer drugs like doxorubicin [41]. MiRNA-21-5p is also linked to tumour development in colorectal cancer, modulating Wnt circuit, RAS-MAPK-PI3K-AKT signalling pathways, as well as TGF-β and p53 components [42]. MiRNA-21 regulated cell proliferation and apoptosis in ESCC through the AKT signalling pathway via targeting cell adhesion molecules [43]. MiRNA-21 has oncogenic properties that play important roles in many cancers, including leukaemia [44]. Multiple data show that miRNA-21 may be an independent prognostic marker for glioma proliferation and invasion, especially those gliomas with high pathological grades, and thus could also be a potential therapeutic target for molecular glioma therapy [45]. MiRNA-21 target sites include a number of genes involved in apoptosis, i.e.: expression of programmed cell death four gene (*PDCD4*) correlates inversely with expression of miRNA-21 in a number of human GBM cell lines such as T98G. Inhibition of miRNA-21 increases endogenous levels of PDCD4 in cell line T98G and overexpression miRNA-21 inhibits PDCD4-dependent apoptosis, functioning as an anti-apoptotic factor [46]. MiRNA-21 exerts its oncogenic function in gliomas through its post-transcriptional targets and downstream signal pathways: PDCD4, phosphatase and tensin homolog (PTEN), tumour protein 63 (TP63), tropomyosin 1 (TPM1), and tissue inhibitor of metalloproteases 3 (TIMP3) [47]. MiRNA-1260a was 0.58-fold and 0.65-fold underexpressed in LN229-GLS(−) and T98G-GLS(−) GBM cells. This data agrees with significant increase in the expression of miRNA-1260a in metastatic tumours as compared to the non-metastatic neuroblastoma in vivo [48]. MiRNA-92a was 0.74-fold and 0.80-fold underexpressed when GLS was silenced in LN229 and T98G cells. Properly, depleting endogenous miRNA-92a-3p inhibits the rate of cell viability, cell migration, and metastasis of U87 and U251 glioma cells in vitro while diminishing tumour volume and weight of xenograft in vivo [49]. Interestingly, miRNA-92a is overexpressed in human GBM cells, including 6-fold in LN229 cells. This research showed that Bim is a direct functional target of miRNA-92a and that high grade gliomas show augmented miRNA-92a levels [50]. MiRNA-92a-3p has showed oncogenic properties, i.e.: increasing cell proliferation, migration and invasion and inhibited cell apoptosis in vitro, as well as augmenting xenograft tumour formation in vivo, in a variety of cancers including leukaemia [44] and gastric [51] cancer. MiRNA-1246 was 2.2-fold and 1.5-fold overexpressed in GLS silenced LN229 and T98G cells, respectively, when compared with control LN229 and T98G cells. These results agree with findings in T98G-GAB(+) vs. T98G-pcDNA cells. In addition, upregulation of miRNA-1246 in prostate cancer significantly decreased growth, augmented apoptosis and inhibited proliferation, invasiveness, and migration in vitro and in vivo [52]. Similar conclusions were obtained in lung cancer [53]. Conversely, miRNA-1246 was overexpressed in MDA-MB-231 breast cancer cells [54].

Concerning antioxidant defence systems, although not similar studies have been found in literature, discordant results have been described, i.e.: antioxidant phenethyl isothiocyanate decreases SOD activity and GSH levels, inducing oxidative stress and repressing LN229 cell growth [55]; but apoptosis induced by cisplatin treatment diminished SOD to impair cancer growth [56]. It has been also reported differences between SOD and CAT responses to oxidative stress and apoptosis in LN229 cells, increasing SOD but decreasing CAT activity [57]. Accordingly, not concluding results were found in our models with reference to SOD and CAT activities (Additional file 5: Fig. S3c, d). Although ROS and antioxidant enzymes show a complex regulation [2], why a different tendency in SOD activity upon GAB overexpression in LN229 as compared to T98G cells is an open question. Impressive, total GSH was established as a clear biomarker of GA-mediated redox regulation: when GLS was silenced GSH levels declined and when GAB was overexpressed GSH content was increased (Additional file 5: Fig. S3a). Similarly, GR activity was another positive biomarker because rose following both GLS silencing and GAB overexpression (Additional file 5: Fig. S3b). Interestingly, GR has been associated to drug resistance in GBM cells, being a potential target for ameliorating GBM treatment [58]. In all models studied in this research, a reduction in both lipid peroxidation and protein damage was found (Additional file 5: Fig. S3e, g), supporting the antioxidant effect of altering GLS and GAB expression and subsequently increasing GR activity (Additional file 5: Fig. S3b). Results depicted in Additional file 5: Fig. S3f strengthen these conclusions: when GLS is silenced in both LN229 and T98G cells TAC is significantly dipped but when GAB was overexpressed in one and the other, LN229 and T98G cells, TAC climbed significantly. Therefore, in spite of scarce studies in GBM cells, changes in oxidative status and antioxidant capacity linked to differential GA expression [3] emerge as promising support against GBM aggressiveness.

In several GBM cell lines, including T98G and LN229, GAB increased the sensitivity towards ROS throughout downregulation of the PI3K/AKT cascade [12]. Changes in oxidative stress through PI3K/AKT linked to miRNA expression levels support our results [59]. Of note, upregulation of miRNA-34a, which is induced by GLS2 through Dicer [17], suppresses antioxidant circuits (SIRT1/PGC-1α/NRF2 pathway) and enhances susceptibility of wild-type p53 cancer cells to ROS [60]. Sirtuin 1 (SIRT1) is involved in the redox balance and carcinogenesis, including the proliferation of GBM cells [61]. Peroxisome proliferator-activated receptor γ coactivator1α (PGC-1α) regulates energy metabolism and oxidative stress, evoking a more aggressive behaviour of GBM cells [62]. Nuclear erythroid factor 2-related factor 2 (NRF2) is a redox-sensitive transcription factor regulating the expression of antioxidant enzymes such as SOD [60].

**Fig. 5:**
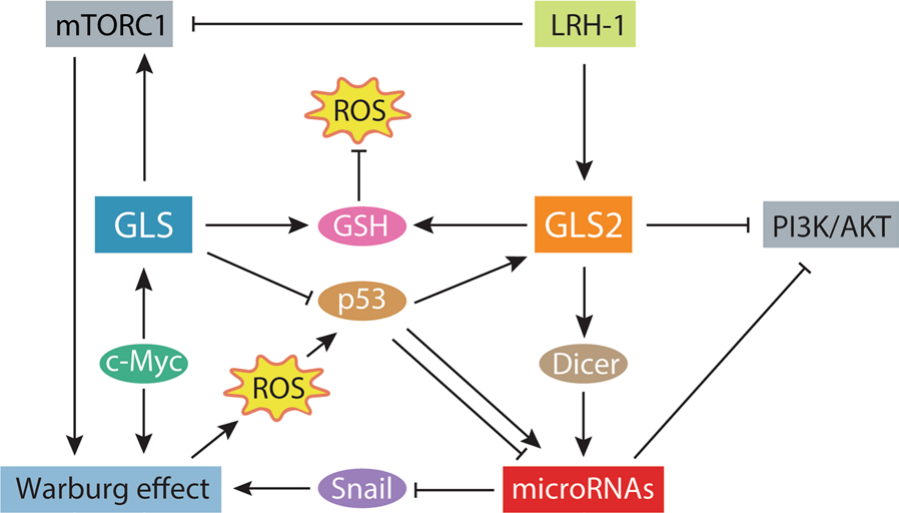
Some metabolic and signalling circuits that intersect with glutaminase isoenzymes. This network schematic representation shows both glutaminase isoenzymes, GLS and GLS2, executing a dual function. First, glutaminase is the limiting enzyme in glutaminolysis to generate glutamate from glutamine, that can be used for GSH synthesis, the most important intracellular antioxidant. Second, both isoenzymes can interact with other proteins and signalling pathways. GLS is regulated by the oncogenic transcription factor c-Myc and activates mTORC1, which is a major positive regulator of the Warburg effect. Tumour suppressor factor p53 is hampered by GLS, but stimulates GLS2. GLS2 blocks PI3K/AKT signalling pathway while interacts and stabilizes Dicer to mature several miRNAs which repress Snail favouring Warburg effect of tumour cells, and impacting oxidative status. P53 connects GLS, GLS2, microRNAs, ROS homeostasis and cancer. *GLS* glutaminase isoenzyme, *GLS2* glutaminase 2 isoenzyme, *GSH *glutathione, *LRH-1* nuclear receptor liver receptor homolog 1, *mTORC1* mammalian target of rapamycin complex 1, *PI3K/AKT *phosphatidylinositol-3-kinase/protein kinase B, *ROS* reactive oxygen specie

On the other hand, GLS2 displays a novel non-metabolic mechanism interacting and stabilizing Dicer protein to thrust miRNA maturation and inhibit Snail expression. These results indicate that GLS2 can act as a suppressor for migration and invasion of cancer cells, repressing EMT [17]. Appropriately, GLS and GLS2 target the expression of Snail through p53 regulation linking GAs to some microRNA expression, which translationally can repress Snail and oncogenic phenotype (Fig. 5). In fact, Snail induces a glycolytic switch to Warburg effect (higher glucose uptake and lactate production) by suppressing mitochondrial oxidative phosphorylation that may contribute to metastasis and tumour progression in breast, colon and ovary cancer [63]. Thus, GA can regulate the expression of some miRNAs, uncovering a previously unsuspected link between glutamine metabolism, miRNAs, ROS homeostasis and cancer, that complement data of other miRNAs regulating GA isoenzymes [11, 15, 16]. Future studies might involve investigating the possibility that GLS silencing and/or GAB overexpression can show synergic effects in combination with miRNA targeting as a new therapeutic strategy against cancer.

## Conclusions

Importantly, this study is the first to identify GA-dependent miRNA expression profiles in glutamine-addicted GBM cells with a consistent pattern of GA expression: high levels of GLS isoforms but only traces or lack of GLS2 transcripts [64]. Noteworthy, miRNA-153 was found to inhibit GBM cell growth in U87MG cell line, modulating GLS expression by directly targeting the 3′-UTR of GLS mRNA [65]. Although some miRNAs have been shown to regulate GA expression in certain types of cancers [66], it is currently unknown whether similar miRNA mechanisms would contribute to GLS activation and GLS2 repression in gliomas [64]. Of interest, a combination therapy which incorporates chemotherapy, radiotherapy, as well as GA- and miRNA-based therapy is likely to be the future direction for the treatment of gliomas [67]. Nonetheless, miRNAs studied in this article will require further functional analysis to evaluate whether they can play a direct role in the clinical outcome of patients suffering from GBM. Second, the marked change in glutathione and oxidative status following GLS silencing or GAB overexpression are a promising tool against cancer progression [68], including glioma cells [69]. Combinatorial and synergic strategies including glutaminase inhibitors are gaining important battles against cancer [70–73]. Isoenzyme specific GA expression [74] and associated miRNAs [75] can contribute to improve the efficiency of new drugs [76] and to block medicament resistance [77], targeting key tumour pathways [78], inducing cancer metabolic reprogramming [79], and helping to win this war in XXI century.

## Supplementary Information

**Additional file 1: Figure S1. **Analysis of GLS protein isoforms (KGA and GAC) downregulation in T98G-GLS(−) and its control (scramble siRNA-transfected) T98G.

**Additional file 2: Figure S2.** MicroRNA expression analysis of T98G(T98G-pcDNA), T98G-GAB(+), LN229 (LN229-GFP), and LN229-GLS(−) cell samples, using miRXplore™ microarrays.

**Additional file 3:** **TableS1.** Steep-loop sequences, mature sequences and probe sequences of the one thousand microRNAs used for microarray experiments.

**Additional file 4:** **TableS2.** The microRNAs ratio list was determined for T98G-GAB(+) vs. T98G(T98G-pcDNA), depicted at column F, and for LN229-GLS(−) vs. LN229 (LN229-GFP) cells, depicted at column G.

**Additional file 5: Figure S3. **Summary of all oxidative stress parameters in the four cell models studied LN229-GLS(−)/LN229 (LN229-GFP), T98G-GLS(−)/T98G (scrambled siRNA-transfected T98G), LN229-GAB(+)/LN229 (LN229-pcDNA) and T98G-GAB(+)/T98G(T98G-pcDNA), with the exception of Protein Damage parameter (g), which shows LN229-GLS(−)/LN229 (LN229-GFP) and T98G-GAB(+)/T98G (T98G-pcDNA).

## Data Availability

All data generated in this study are available from the corresponding author on reasonable request.

## Abbreviations

AKT: Protein kinase B
ATCC: American Type Culture Collection
CAT: Catalase
CRE: Copper Reducing Equivalents
DMEM: Dulbecco's Modified Eagle Medium
DTNB: 5,5′-dithiobis-(2-nitrobenzoic acid)
EGFR: Epidermal growth factor receptor
EMT: Epithelial-mesenchymal transition
ESCC: Oesophageal squamous cell carcinoma
FBS: Foetal bovine serum
FTC: Fluorescein 5-thiosemicarbazide
GA: Glutaminase
GAB: Longer GLS2 transcript
GAC: Shorter GLS transcript
GFP: Green fluorescent protein
GBM: Glioblastoma
GLS: Glutaminase isoenzyme
GLS2: Glutaminase isoenzyme 2
GR: Glutathione reductase
GSSG: Oxidized glutathione
GSH: Reduced glutathione
HCC: Hepatocellular carcinoma
KGA: Longer GLS transcript
LGA: Shorter GLS2 transcript
lncRNA: Long non-coding ribonucleic acid
MAPK: Mitogen-activated protein kinase
MDA: Malondialdehyde
miRNA: microRNA
mTOR: Mammalian target of rapamycin
NRF2: Nuclear erythroid factor 2-related factor 2
OSCC: Oral squamous cell carcinoma
PDCD4: Programmed cell death four gene
PDGFRB: Platelet-derived growth factor receptor beta
PGC-1α: Peroxisome proliferator-activated receptor γ coactivator1α
PI3K: Phosphatidylinositol-3-kinase
PTEN: Phosphatase and tensin homolog
qRT-PCR: Quantitative reverse transcription-polymerase chain reaction
RIN: RNA integrity numbers
RNAi: RNA interference
ROS: Reactive oxygen species
SD: Standard deviation
siRNA: Small interferent RNA
SIRT1: Sirtuin 1
SOD: Superoxide dismutase
TAC: Total antioxidant capacity
TBARS: Thiobarbituric acid reactive substances
tGSH: Total glutathione
TGF-β: Transforming growth factor-β
TIMP3: Tissue inhibitor of metalloproteases 3
TP63: Tumour protein 63
TPM1: Tropomyosin 1
UCA1: Urothelial carcinoma-associated 1
