# Supplementary figures and images for: Glutaminase isoforms expression switches microRNA levels and oxidative status in glioblastoma cells
Source: J Biomed Sci. 2021 Feb 20;28:14. doi: 10.1186/s12929-021-00712-y (PMC7897386; doi:10.1186/s12929-021-00712-y)

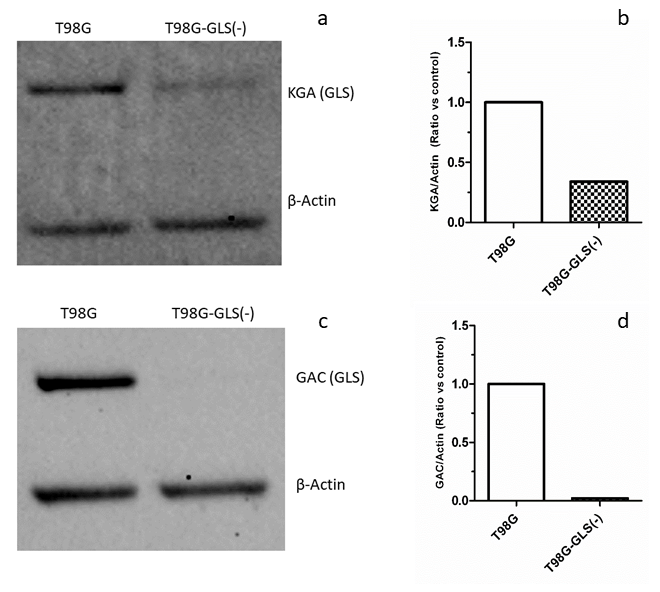

Supplement: Supplementary file 1 — Additional file 1: Figure S1. Analysis of GLS protein isoforms (KGA and GAC) downregulation in T98G-GLS(−) and its control (scramble siRNA-transfected) T98G. [file 12929_2021_712_MOESM1_ESM.bmp]

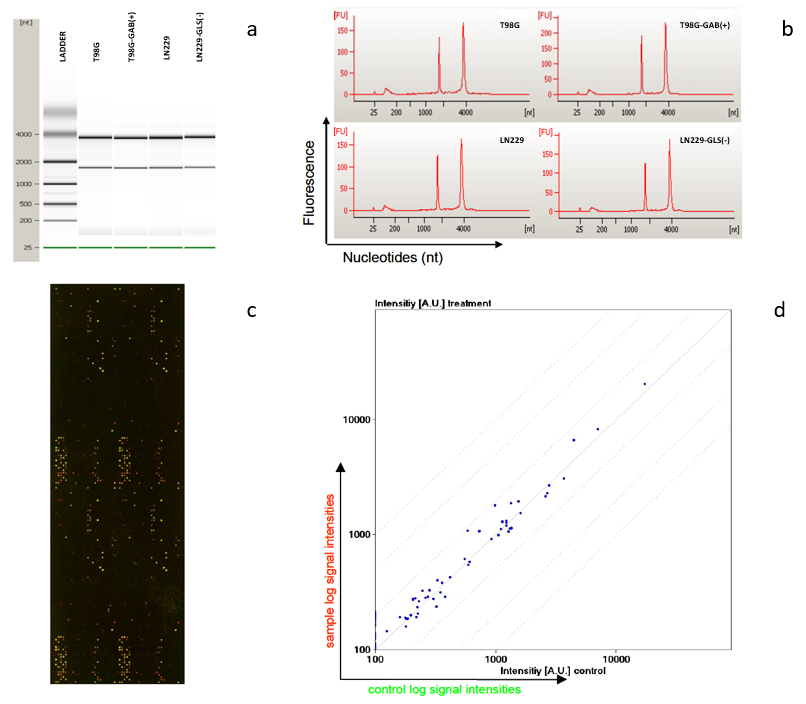

Supplement: Supplementary file 2 — Additional file 2: Figure S2. MicroRNA expression analysis of T98G(T98G-pcDNA), T98G-GAB(+), LN229 (LN229-GFP), and LN229-GLS(−) cell samples, using miRXplore™ microarrays. [file 12929_2021_712_MOESM2_ESM.bmp]
